# The Prevalence of Mild Cognitive Impairment in Diverse Geographical and Ethnocultural Regions: The COSMIC Collaboration

**DOI:** 10.1371/journal.pone.0142388

**Published:** 2015-11-05

**Authors:** Perminder S. Sachdev, Darren M. Lipnicki, Nicole A. Kochan, John D. Crawford, Anbupalam Thalamuthu, Gavin Andrews, Carol Brayne, Fiona E. Matthews, Blossom C. M. Stephan, Richard B. Lipton, Mindy J. Katz, Karen Ritchie, Isabelle Carrière, Marie-Laure Ancelin, Linda C. W. Lam, Candy H. Y. Wong, Ada W. T. Fung, Antonio Guaita, Roberta Vaccaro, Annalisa Davin, Mary Ganguli, Hiroko Dodge, Tiffany Hughes, Kaarin J. Anstey, Nicolas Cherbuin, Peter Butterworth, Tze Pin Ng, Qi Gao, Simone Reppermund, Henry Brodaty, Nicole Schupf, Jennifer Manly, Yaakov Stern, Antonio Lobo, Raúl Lopez-Anton, Javier Santabárbara, Cohort Studies of Memory in an International Consortium (COSMIC)

**Affiliations:** 1 Centre for Healthy Brain Ageing, University of New South Wales, Sydney, Australia; 2 Dementia Collaborative Research Centre, University of New South Wales, Sydney, Australia; 3 Department of Public Health and Primary Care, Cambridge University, Cambridge, United Kingdom; 4 MRC Biostatistics Unit, Institute of Public Health, Cambridge, United Kingdom; 5 Institute of Health and Society, Newcastle University, Newcastle upon Tyne, United Kingdom; 6 Saul B. Korey Department of Neurology, Albert Einstein College of Medicine, Yeshiva University, New York City, New York, United States of America; 7 Department of Epidemiology and Population Health, Albert Einstein College of Medicine, Yeshiva University, New York City, New York, United States of America; 8 Inserm, U1061 Nervous System Pathologies: Epidemiological and Clinical Research, La Colombière Hospital, Montpellier Cedex 5, France; 9 Université de Montpellier 1, Montpellier, France; 10 Faculty of Medicine, Imperial College, St Mary’s Hospital, London, United Kingdom; 11 Department of Psychiatry, The Chinese University of Hong Kong, Hong Kong Special Administrative Region, China; 12 Department of Psychiatry, Tai Po Hospital, Hong Kong Special Administrative Region, China; 13 GolgiCenci Foundation, Abbiategrasso (Milan), Italy; 14 Department of Psychiatry, University of Pittsburgh School of Medicine, Pittsburgh, Pennsylvania, United States of America; 15 Department of Neurology, University of Pittsburgh School of Medicine, Pittsburgh, Pennsylvania, United States of America; 16 Department of Epidemiology, University of Pittsburgh Graduate School of Public Health, Pittsburgh, Pennsylvania, United States of America; 17 Department of Neurology, Oregon Health and Science University, Portland, Oregon, United States of America; 18 Department of Neurology, University of Michigan, Ann Arbor, Michigan, United States of America; 19 Centre for Research on Ageing, Health and Wellbeing, College of Medicine, Biology and Environment, The Australian National University, Canberra, Australia; 20 Gerontology Research Programme, Department of Psychological Medicine, Yong Loo Lin School of Medicine, National University of Singapore, Singapore, Singapore; 21 The Taub Institute for Research in Alzheimer’s Disease and the Aging Brain, Columbia University, New York City, New York, United States of America; 22 The Gertrude H. Sergievsky Center, Columbia University, New York City, New York, United States of America; 23 The Division of Epidemiology, Joseph P. Mailman School of Public Health, Columbia University, New York City, New York, United States of America; 24 The Department of Neurology, Columbia University, New York City, New York, United States of America; 25 Centro de Investigación Biomédica en Red de Salud Mental (CIBERSAM), Ministry of Science and Innovation, Madrid, Spain; 26 Department of Medicine and Psychiatry, Universidad de Zaragoza, Zaragoza, Spain; 27 Centro de Investigación Biomédica en Red de Salud Mental, CIBERSAM, Spanish Ministry of Economy and Competitiveness, Madrid, Spain; 28 Department of Microbiology, Preventive Medicine and Public Health, University of Zaragoza, Zaragoza, Spain; University of Leipzig, GERMANY

## Abstract

**Background:**

Changes in criteria and differences in populations studied and methodology have produced a wide range of prevalence estimates for mild cognitive impairment (MCI).

**Methods:**

Uniform criteria were applied to harmonized data from 11 studies from USA, Europe, Asia and Australia, and MCI prevalence estimates determined using three separate definitions of cognitive impairment.

**Results:**

The published range of MCI prevalence estimates was 5.0%–36.7%. This was reduced with all cognitive impairment definitions: performance in the bottom 6.681% (3.2%–10.8%); Clinical Dementia Rating of 0.5 (1.8%–14.9%); Mini-Mental State Examination score of 24–27 (2.1%–20.7%). Prevalences using the first definition were 5.9% overall, and increased with age (*P* < .001) but were unaffected by sex or the main races/ethnicities investigated (Whites and Chinese). Not completing high school increased the likelihood of MCI (*P* ≤ .01).

**Conclusion:**

Applying uniform criteria to harmonized data greatly reduced the variation in MCI prevalence internationally.

## Introduction

Mild cognitive impairment (MCI) refers to cognitive decline from a previous level of functioning, both subjectively and by objective evidence. The condition was first conceptualized in the 1980s, at which time it focused on memory impairment and was thought to be a transitional stage between normal cognitive ageing and Alzheimer’s disease (AD) [1], and assigned to corresponding stages on both the Clinical Dementia Rating (CDR; rating of 0.5) [2] and Global Deterioration Scale (stages 2 and 3) [3]. While individuals with MCI due to AD may go on to develop dementia due to AD, others with MCI may develop different subtypes of dementia [4–7]. It has also become apparent that MCI is not necessarily a pre-dementia syndrome, as many individuals with MCI do not show progression of their cognitive deficits, and may in some cases revert to normal cognition [6, 8, 9]. Accordingly, the concept of MCI has evolved to accommodate heterogeneity in outcomes and aetiologies, in part through the development of MCI subtypes with greater potential clinical and prognostic value [1, 10]. The major MCI subtypes are amnestic (aMCI), involving episodic memory impairment (with or without impairment in other cognitive domains), and non-amnestic (naMCI), involving impairment in cognitive domains other than memory. Of these subtypes, aMCI is considered more likely to progress to AD, and naMCI more likely to progress to other types of dementia [10].

The level of cognitive decline associated with MCI is greater than expected for age, but not as severe as that associated with dementia. Another point of difference is retention of independence in activities of daily living (ADLs) with MCI [11, 12]. This can be difficult to establish however, as people with MCI can show impairments in high level or complex functional tasks [11, 13]. A decrease in the ability to perform such tasks can predict a decline from MCI to dementia [8].

Since MCI imposes a health burden of its own and increases the risk of dementia, it is important to reliably estimate the prevalence of MCI around the globe. However, a recent review found that the reported prevalence of MCI varies widely across international studies, from around 3% to 42% [14]. This high level of variability in reported MCI prevalence poses problems for public health policy and planning. Some of the variation may be associated with regional and/or ethnic differences. For example, the prevalence of aMCI in India is reportedly more than five times higher than in China, despite standardization for age, sex and education [15]. Another study found a higher prevalence of naMCI in Blacks than in Whites from a similar geographical location, even when sex and education were controlled [16]. That study also found the prevalence of aMCI to increase with age among men and blacks. Others have found that the prevalence of MCI increased with age [17, 18], or that men had a higher prevalence of either MCI [18] or aMCI [19]. Education also appears to influence the prevalence of MCI [17]. While findings like these suggest that differences in location and demographic make-up may contribute to the wide variation in reported prevalence of MCI, a significant proportion can be attributed to differences in definition and methodology [14]. For example, studies have not been consistent in how they have defined objective cognitive impairment. Small changes to elements of this criterion, such as the threshold for impairment and the number of sub-threshold cognitive test results required, can greatly affect the prevalence of MCI found [20]. A further issue is the use of only global scales by many studies, with limited forms of neuropsychological testing less likely than comprehensive test batteries to reliably identify MCI [21].

We have recently developed an international consortium—Cohort Studies of Memory in an International Consortium (COSMIC) [22]–which has brought together data from cohort studies of cognitive aging internationally. The goal of this study was to harmonize these data and apply uniform diagnostic criteria to more reliably estimate MCI prevalence across different geographical and ethnocultural regions. We present data from three studies in USA, four in Europe, two in Asia, and two in Australia.

## Materials and Methods

### Contributing studies and participants

Cross-sectional analyses of 11 longitudinal population-based studies of cognitive aging (listed in Table 1, with abbreviations) were performed. Rather than the full population of each study, we used samples comprising individuals aged 60 or more years who were not identified as having dementia and/or did not have a CDR [34] ≥ 1. Any individuals with missing age, sex or dementia status data were excluded. A number of samples did not require exclusions for dementia because individuals with dementia were already omitted during the recruitment phase of the study. Table 2 shows the demographic characteristics of the samples used in our analyses, including the main race or ethnicity represented (White in 7 studies and Chinese in 2 studies). As a project of the COSMIC collaboration, the present study was performed with approval from the University of New South Wales Human Research Ethics Committee (Ref: # HC12446). Each of the 11 extant studies contributing data to the present study had previously obtained ethics approval from their respective institutional review boards, and all participants within the studies provided consent (for details see the references listed in Table 1). The particular use of these data for the present study did not warrant further participant consent, with de-identified health data not considered to be protected health information under current research principles (e.g., as per the Privacy Rule proposed by the National Institute of Health, USA http://privacyruleandresearch.nih.gov/research_repositories.asp).

**Table 1:** Contributing studies.

Study	Abbreviation	Country	Reference
Cognitive Function & Ageing Studies	CFAS	UK	Brayne et al. [23]
Einstein Aging Study	EAS	USA	Katz et al. [16]
Etude Santé Psychologique Prévalence Risques et Traitement	ESPRIT	France	Ritchie et al. [24]
Hong Kong Memory and Ageing Prospective Study	HK-MAPS	Hong Kong	Wong et al. [25]
Invecchiamento Cerebrale in Abbiategraso	Invece.Ab	Italy	Guaita et al. [26]
Monongahela Valley Independent Elders Survey	MoVIES	USA	Ganguli et al. [27]
Personality and Total Health Through Life Project	PATH	Australia	Anstey et al. [28]
Singapore Longitudinal Ageing Studies	SLAS	Singapore	Feng et al. [29, 30]
Sydney Memory and Ageing Study	Sydney MAS	Australia	Sachdev et al. [31]
Washington Heights Inwood and Columbia Aging Project	WHICAP	USA	Tang et al. [32]
Zaragoza Dementia Depression Project	ZARADEMP	Spain	Lobo et al. [33]

**Table 2:** Sample characteristics.

Characteristic	CFAS	EAS	ESPRIT	HK-MAPS	Invece.Ab	MoVIES	PATH	SLAS	Sydney MAS	WHICAP	ZARADEMP
	(N = 2050)	(N = 1954)	(N = 2189)	(N = 786)	(N = 1267)	(N = 1276)	(N = 1973)	(N = 3950)	(N = 1037)	(N = 3991)	(N = 4415)
Age (yrs)											
Mean ± SD	75.8 ± 7.3	78.3 ± 5.4	73.1 ± 5.6	72.3 ± 7.2	71.2 ± 1.3	74.2 ± 5.4	70.6 ± 1.5	68.5 ± 6.3	78.8 ± 4.8	76.4 ± 6.5	73.4 ± 9.3
Range	64–105	63–100	65–96	60–96	70–75	66–97	68–74	60–97	70–90	63–103	60–102
Sex											
Female	1289 (62.9)	1191 (61.0)	1277 (58.3)	422 (53.7)	684 (54.0)	775 (60.7)	953 (48.3)	2382 (60.3)	572 (55.2)	2684 (67.3)	2518 (57.0)
Male	761 (37.1)	763 (39.0)	912 (41.7)	364 (46.3)	583 (46.0)	501 (39.3)	1020 (51.7)	1568 (39.7)	465 (44.8)	1307 (32.7)	1897 (43.0)
Education											
Less than completed high school	195 (9.5)	428 (21.9)	550 (25.1)	686 (87.3)	1150 (90.8)	500 (39.2)	466 (23.6)	3449 (87.3)	437 (42.1)	2163 (54.2)	3838 (86.9)
Completed high school	1399 (68.2)	906 (46.4)	911 (41.6)	49 (6.2)	90 (7.1)	544 (42.6)	202 (10.2)	73 (1.8)	177 (17.1)	733 (18.4)	128 (2.9)
Technical/college diploma	286 (14.0)	97 (5.0)	210 (9.6)	8 (1.0)	NA^a^	193 (15.1)	610 (30.9)	265 (6.7)	112 (10.8)	480 (12.0)	281 (6.4)
University degree	148 (7.2)	478 (24.5)	516 (23.6)	42 (5.3)	27 (2.1)	39 (3.1)	674 (34.2)	161 (4.1)	311 (30.0)	605 (15.2)	131 (3.0)
Missing data	22 (1.1)	45 (2.3)	2 (0.1)	1 (0.1)	0 (0.0)	0 (0.0)	21 (1.1)	2 (0.1)	0 (0.0)	10 (0.3)	37 (0.8)
Race/ethnicity	White	White (67%); Black (27%)	White	Chinese	White	White	White	Chinese	White	Hispanic (48%); Black (30%); White (21%)	White

NA = not applicable. Data are n (%) unless stated otherwise. N is for the contributed sample that includes individuals from the full study sample without dementia and with no missing data on the baseline dementia, age and sex variables. Age corresponds to the assessment wave for which data were provided: baseline for all studies except MoVIES (wave 2) and PATH (wave 3). For race/ethnicity, the sample was comprised entirely or predominantly of that shown unless otherwise indicated.

^a^ Technical/college diploma could not be distinguished from Completed high school for Invece.Ab.

### Measures and harmonization

Of the 11 contributing studies, nine provided raw data needed to make classifications of MCI (summarized in S1–S5 Tables). The Sydney team processed these data, which were harmonized, when necessary, and pooled. The other two studies, CFAS and Invece.Ab, conducted analyses in-house using the protocols developed for this report. Baseline or wave 1 data were used for all studies except two that did not have all variables needed to make MCI classifications until later waves: MoVIES [27] provided data for wave 2 (2 years after baseline), and PATH [28] for wave 3 (8 years after baseline). Data for SLAS are for two cohorts, SLAS-1 recruited 2003–2004 and SLAS-2 recruited 2008–2011, and for whom the same core measurements and procedures were used [29, 30].

### Demographics

Information included age, sex and education. Education data were harmonized by forming a variable with four categories: Less than high school completion; high school completion; technical or college diploma; university degree (as shown in S6 Table). Note that we use the term college diploma in an international context, referring to courses of less duration and standard than a bachelor’s degree that are typically provided by technical, applied, or more vocationally oriented institutions rather than universities. Each of the contributing studies helped determine how the data representing their local education system was best transformed to the four categories we used.

### Functional ability

A variety of instruments assessing ADLs and instrumental ADLs (IADLs) was used across the studies (S7 Table), with the Lawton & Brody IADL Scale [35] used by five studies. Six common and compatible IADL items were chosen for harmonization: telephone, food preparation, medications, shopping, finances, and transport. For each of these items, the Lawton & Brody IADL Scale assigns a score of 0 to dependent responses and 1 to independent responses. We produced a dichotomized variable of 0 (dependence) and 1 (independence) for each item by matching responses from different instruments to this scoring system. The strictness or level of assistance required for a classification of dependence varies between items on the Lawton & Brody Scale, and thus a response from another instrument such as “Yes, some difficulty” could be considered independent on one of the harmonized items but dependent on another. For full details of this procedure see S8 and S9 Tables.

### Cognitive ability or status

All studies, except for ZARADEMP, administered a neuropsychological test battery; additionally, all except for EAS and WHICAP used the Mini-Mental State Examination (MMSE). However, EAS administered the Blessed Information-Memory-Concentration test, and a validated formula was used to convert scores for this test to MMSE scores [36]. More than half of the studies administered the CDR Scale (Table 3). There was limited overlap in the neuropsychological tests used between studies, and each test score was allocated to one of five cognitive domains: memory, attention/processing speed, language, executive function, and perceptual-motor. However, the perceptual-motor domain was not used in classifying MCI as scores could not be formed for four studies (ESPRIT, HK-MAPS, Invece.Ab, and PATH). Tests were allocated to domains to be consistent with common practice (as outlined in the S1 Text and S10–S14Tables) [37–39]. Domain scores were calculated separately for each study, using information from within the study only. The first step was to adjust test scores for age, sex and education, and for all interactions between these variables using regression analyses. Such adjustments are standard practice in neuropsychological assessment because these variables may significantly affect test performance [37]. Further, the use of age and education adjusted norms has been particularly recommended when assessing the objective cognitive impairment criterion for MCI [11]. Our use of these adjustments should therefore have yielded prevalences for MCI similar to those found were each study to independently make new classifications of MCI using the same set of recent international guidelines. The adjusted test scores were then transformed to Z-scores using the mean and SD of the study sample as normative values. It has been argued and shown that more equivalent and accurate comparisons of cognitive performance between countries are facilitated by the use of country-specific norms [40]. However, for many countries, including those represented by the studies contributing to this investigation, there are no published normative data available. Indeed, a survey of the COSMIC member studies shows that even when norms for particular cognitive tests are available they are often not age-appropriate for elderly samples. Thus, the largely incomplete availability of external norms helped determine our choice to use study-specific internal norms. After forming Z-scores for the tests on the basis of these internal norms, composite scores for each domain were calculated as the mean of the Z-scores of the relevant component tests. These composite scores were themselves then transformed to Z-scores to ensure that all domains had means of 0 and SDs of 1 within each study. Full details and reasons for how domain scores were calculated are in S1 Text.

**Table 3:** Prevalence of mild cognitive impairment: subtypes, and as based on Clinical Dementia Ratings and Mini-Mental State Examination scores.

	aMCI		naMCI		MMSE		CDR	
Study	Crude	Standardized	Crude	Standardized	Crude	Standardized	Crude	Standardized
CFAS	NA	NA	NA	NA	11.3 (9.1–13.9)	13.9 (11.7–16.1)	NA	NA
EAS	1.8 (1.3–2.6)	1.4 (0.9–1.9)	2.6 (1.9–3.4)	2.0 (1.4–2.6)	10.3 (8.9–11.9)	6.7 (5.7–7.7)	6.6 (5.4–8.0)	5.2 (4.1–6.2)
ESPRIT	1.2 (0.8–1.7)	1.3 (0.7–1.8)	3.5 (2.8–4.4)	3.7 (2.8–4.5)	9.2 (8.1–10.6)	9.4 (8.0–10.7)	NA	NA
HK-MAPS	1.0 (0.4–2.6)	0.5 (0.0–0.9)	4.4 (2.9–6.9)	5.2 (2.7–7.6)	14.4 (11.8–17.3)	14.5 (11.7–17.3)	13.6 (11.3–16.2)	14.9 (12.1–17.8)
Invece.Ab	3.9 (3.0–5.2)	3.0 (2.3–3.7)	4.4 (3.3–5.8)	4.0 (3.1–4.9)	10.7 (9.0–12.7)	9.9 (8.5–11.2)	NA	NA
MoVIES	2.6 (1.9–3.6)	2.6 (1.7–3.5)	4.7 (3.6–6.0)	5.1 (3.8–6.4)	18.5 (16.4–20.8)	19.1 (16.8–21.4)	1.5 (1.0–2.3)	1.8 (1.0–2.6)
PATH	1.0 (0.6–1.6)	1.0 (0.6–1.5)	2.3 (1.6–3.1)	2.4 (1.7–3.0)	1.3 (0.9–2.0)	2.1 (1.5–2.7)	NA	NA
SLAS	2.0 (1.4–2.9)	2.2 (1.3–3.1)	3.2 (2.4–4.2)	2.8 (1.7–3.9)	6.5 (5.5–7.6)	6.7 (5.5–8.0)	13.6 (11.3–16.3)	13.9 (10.5–17.3)
Sydney MAS	4.0 (2.9–5.5)	3.6 (2.5–4.7)	6.7 (5.3–8.6)	6.2 (4.7–7.6)	16.7 (14.4–19.3)	17.4 (15.1–19.6)	9.2 (7.4–11.3)	9.6 (7.8–11.3)
WHICAP	1.6 (1.3–2.1)	1.5 (1.1–1.9)	4.8 (4.0–5.6)	4.6 (3.8–5.4)	NA	NA	9.5 (8.6–10.5)	9.3 (8.3–10.2)
ZARADEMP	NA	NA	NA	NA	19.7 (18.4–21.1)	20.7 (19.2–22.1)	NA	NA
Total	2.0 (1.7–2.2)	2.0 (1.7–2.2)	3.9 (3.6–4.2)	3.9 (3.6–4.2)	12.0 (11.5–12.5)	12.0 (11.5–12.4)	8.5 (8.0–9.2)	9.0 (8.4–9.6)

aMCI = amnestic mild cognitive impairment; naMCI = non-amnestic mild cognitive impairment; CDR = Clinical Dementia Rating; MMSE = Mini-Mental State Examination; NA = not applicable. Values are percentage prevalence (95% confidence interval). The objective cognitive impairment criteria for these classifications was for aMCI: performance in the bottom 6.681% of the relevant study for the memory domain; naMCI: performance in the bottom 6.681% of the relevant study for at least one harmonized cognitive domain other than and excluding memory; CDR: a rating of 0.5; MMSE: a score of 24–27. Standardized prevalence estimates were directly standardized for age group and sex, with the standard population being the total sample of all studies included in the analysis; data were imputed for the missing age ranges within Invece.Ab, PATH and Sydney MAS.

### Subjective cognitive complaint or concern

The means by which the contributing studies ascertained the presence or absence of a cognitive complaint or concern varied widely. Some studies asked a single question of the participant, while others used different and sometimes multiple approaches that included clinical impression, standardized instruments, and informant reports. We attempted to minimize any biases associated with these differences, particularly those related to having different numbers of opportunities to endorse a complaint, by choosing to use only the most generalized question asked of the participant (either in isolation or extracted from a longer instrument) from each study (see S15 Table). This was also the default option given that some studies only had one relevant item. All of the questions addressed memory only, except those used by SLAS, which also addressed thinking or other mental abilities. We dichotomized the responses to indicate either the presence or absence of a subjective cognitive complaint or concern.

### Classification of MCI

MCI was classified using the four generally accepted criteria: absence of dementia, no or minimal functional impairment, subjective memory/cognitive complaint or concern, and objective cognitive impairment [12]. Most studies reported making classifications of dementia using DSM-IV criteria (see S16 Table). Note that a number of studies excluded any individuals with dementia at the recruitment stage of their study. Functional impairment was defined as dependence in two or more harmonized IADL items (the handling of missing data is detailed in S1 Text). Cognitive complaints or concerns were determined as above. Objective cognitive impairment for each cognitive domain was a score within the bottom 6.681% of the scores for that domain within the relevant study’s sample, which is the equivalent of impairment being defined as scores more than 1.5 SDs below the mean. A classification of MCI required impairment in any of the four domains used. Impairment on the memory domain was needed for aMCI, and impairment on any of the other domains (without memory impairment) for naMCI; individuals with data for fewer than three of the non-memory domains were included if impairment was present in one or more domain, otherwise excluded. For other analyses, objective cognitive impairment was defined as an MMSE score from 24 to 27 (inclusive), or as a CDR of 0.5. An MMSE score of 24–27 has been previously used to define a range of milder forms of cognitive impairment, including MCI [41, 42], mild cognitive decline [43] and mild AD [44], and a CDR of 0.5 is a commonly-used criteria for MCI (e.g., [45, 46]).

### Statistical analysis

The Sydney team performed all analyses of the harmonized and pooled data. Five different MCI classifications were made: MCI and its aMCI, and naMCI subtypes using harmonized cognitive domain scores for determining objective cognitive impairment; and MCI using each of MMSE and CDR scores for this criterion. For each type of classification, only individuals with complete data for all four MCI criteria were included. Crude prevalence was determined for men and women in three age groups (60–69, 70–79 and 80–89 yrs). The Wilson score method described, evaluated and endorsed by Newcombe [47] was used to determine 95% confidence intervals (CIs), and chi-square tests were used to make comparisons. Prevalence estimates and CIs directly standardized for age and sex were calculated (for details see S1 Text). For studies with participants in only one or two of the age groups, data for the remaining age group or groups were generated using a multiple imputation procedure (outlined in S1 Text). Associations between educational level and MCI were investigated with logistic regressions that controlled for age and sex. The two studies that provided results rather than raw data (CFAS and Invece.Ab) used the standard protocols developed for this report. The analyses were done using IBM SPSS Statistics 20, and the imputations done using the R-package mice 2.21.

## Results

### Sample description

The demographic characteristics of the cohorts are provided in Table 2. The samples varied in size from 786 (HK-MAPS) to 4,415 (ZARADEMP), with a median of 2,000, and total sample of 24,888 (59.3% women; mean age 73.6 yrs). The purpose and design of both Invece.Ab [26] and PATH [28] led to them having narrower age cohorts than the other contributing studies. The sample sizes for each approach for classifying MCI are shown in S17 Table.

### Prevalence of MCI

**Fig 1:**
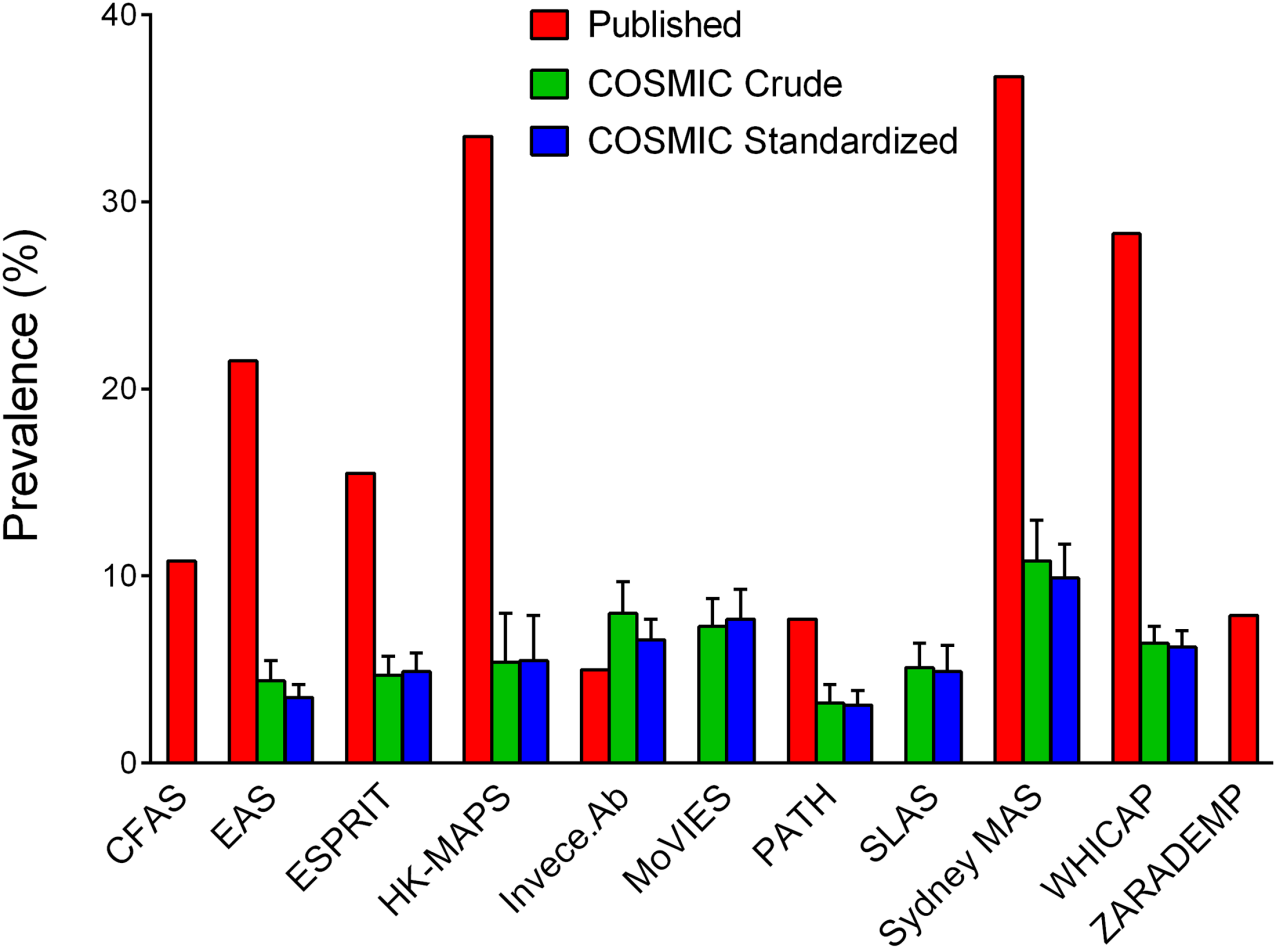
Prevalence estimates of mild cognitive impairment previously published and as obtained using COSMIC protocols. Error bars indicate the upper limits of 95% confidence intervals. For the crude and standardized prevalence estimates obtained using COSMIC protocols, the criterion for objective cognitive impairment was performance in the bottom 6.681% for the study on at least one harmonized cognitive domain. Estimates were directly standardized for age and sex, with the standard population being the total sample of all studies included in the analysis; data were imputed for missing age ranges within Invece.Ab, PATH and Sydney MAS. SLAS had not previously published prevalence estimates of mild cognitive impairment (MCI), and a published estimate for MoVIES was for amnestic MCI only [19]. CFAS and ZARADEMP did not have neuropsychological test data from which harmonized cognitive domain scores could be derived. Published prevalence estimates are for baseline, except for PATH (wave 3, the first assessment when all relevant data for classifying MCI were obtained). References for the published estimates shown are: CFAS [48]; EAS [16]; ESPRIT [24]; HK-MAPS [25]; Invece.Ab [49]; PATH [50]; Sydney MAS [51]; WHICAP [52]; ZARADEMP [53]. Note that the HK-MAPS sample was over-represented by individuals considered to be at increased risk of conversion to dementia, and the prevalence estimates for MCI shown are likely to overestimate those for the broader population [25].

**Fig 2:**
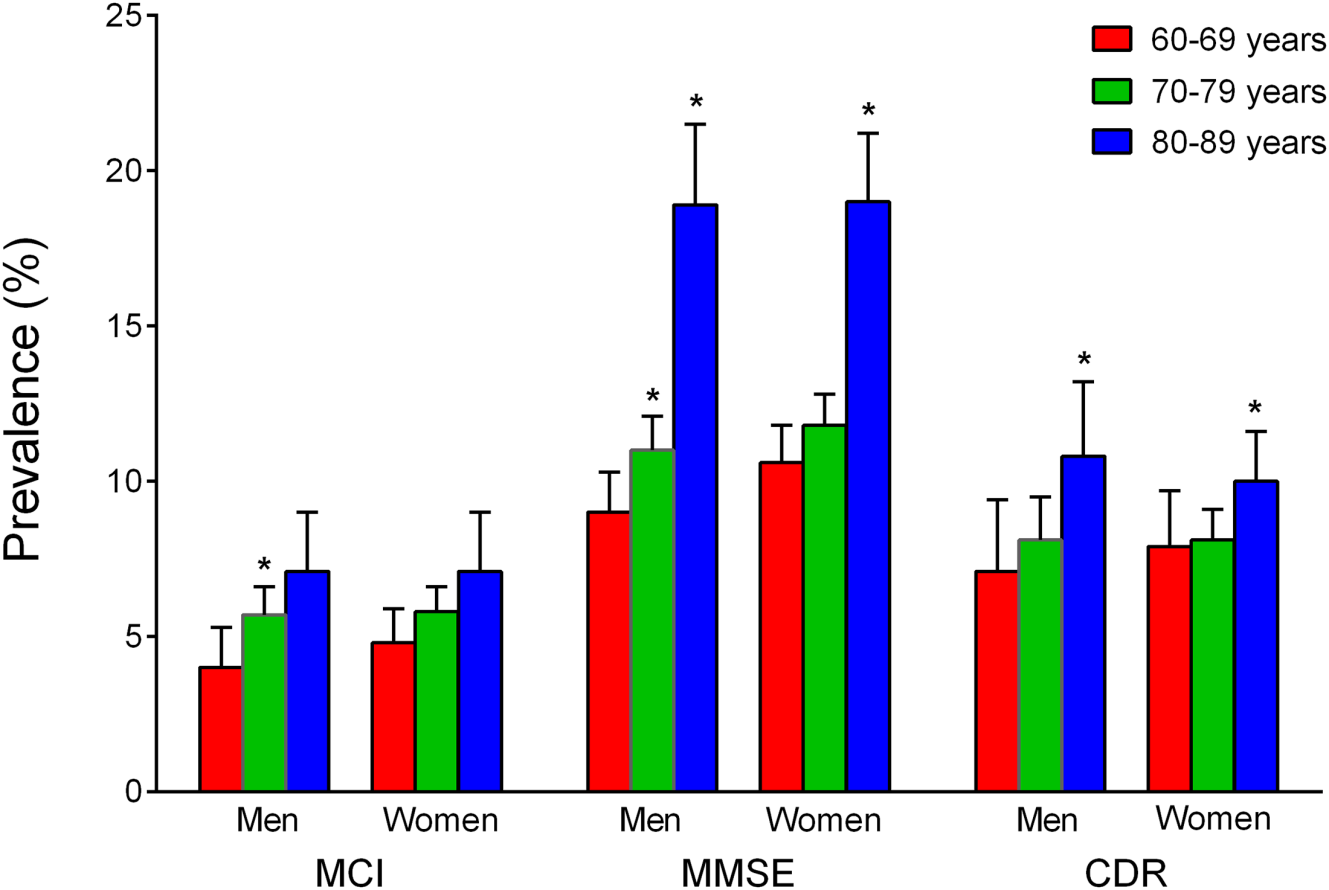
Crude prevalence estimates of mild cognitive impairment (MCI) among men and women of different age groups. Error bars indicate upper limits of 95% confidence intervals. Asterisks indicate a significant difference from: * 60–69 years; ** 70–79 years. There were no significant differences between men and women of the same age group for any classification approach. The objective cognitive impairment criteria for the classifications was performance in the bottom 6.681% of the relevant study for at least one harmonized cognitive domain (mild cognitive impairment), a Clinical Dementia Rating (CDR) of 0.5, or a Mini-Mental State Examination (MMSE) score 24–27.

Fig 1 shows the prevalences of MCI previously published by the contributing studies, alongside the crude and standardized prevalences obtained using COSMIC protocols that defined cognitive impairment as performance in the bottom 6.681%. With this criterion, the crude prevalence was 5.9 (5.5–6.3)% overall, and increased with age: from 4.5% among 60–69 year-olds to 5.8% among 70–79 year-olds (χ^2^ = 6.80, df = 1, *P* = .009), and to 7.1% among 80–89 year-olds (χ^2^ = 5.28, df = 1, *P* = .022 vs. 70–79 year-olds). The crude prevalence for men was higher among 70–79 year-olds than among 60–69 year-olds (χ^2^ = 4.62, df = 1, *P* = .032), but not significantly higher among 80–89 year-olds than 70–79 year-olds (see Fig 2). For women, the only increase was from 60–69 year-olds to 80–89 year-olds (χ^2^ = 7.82, df = 1, *P* = .005). There were no significant differences between men and women within any of the age groups. The average age- and sex-standardized prevalence for 60–89 year-olds was 5.8 (5.4–6.2)%. The standardized prevalence differed across the studies (χ^2^ = 94.64, df = 8, *P* < .001), but not between Chinese (5.2%, 95% CI = 4.1–6.4%) and Whites (5.8%, 5.3–6.3%; χ^2^ = 0.76, df = 1, *P* = .383).

Higher figures were obtained when MMSE scores 24–27 were used as the criterion, with an overall total crude prevalence of 12% (see Table 3). Crude prevalence estimates were greater among 70–79 year-olds than 60–69 year-olds for men (χ^2^ = 5.80, df = 1, *P* = .016), and greater among 80–89 year-olds than 70–79 year-olds for both men (χ^2^ = 43.10, df = 1, *P* < .001) and women (χ^2^ = 46.07, df = 1, *P* < .001; Fig 2). There were no significant differences between men and women within any of the age groups. Standardized prevalence estimates were significantly higher for White populations (12.9%, 12.3–13.4%) than for Chinese (8.6%, 7.4–9.7%; χ^2^ = 42.34, df = 1, *P* < .001). All studies except WHICAP had MMSE scores, and it is noteworthy that the Spanish study (ZARADEMP), which only had MMSE scores, yielded the highest crude and standardized prevalence figures using this measure, thereby influencing the overall prevalence estimates. The CDR, administered by only six studies, yielded prevalence estimates of 8.5% (overall crude) and 9.0% (overall standardized), which were in between those based on MMSE scores and those based on harmonized cognitive domain scores (see Table 3). CDR-based prevalence estimates were significantly greater among 80–89 year-olds than 70–79 year-olds for both men (*χ* ^2^ = 4.74, df = 1, *P* = .030) and women (*χ* ^2^ = 4.46, df = 1, *P* = .035; Fig 2). The correspondence between the prevalence estimates with MMSE or CDR was low.

The range of MCI prevalence estimates published by the contributing studies was 5.0%–36.7% (see Fig 1). This considerable variation in estimates was reduced with all three of the definitions for cognitive impairment that we used: performance in the bottom 6.681% (3.2%–10.8%); CDR of 0.5 (1.8%–14.9%); MMSE score of 24–27 (2.1%–20.7%).

### Prevalence of MCI subtypes

The overall crude prevalence of aMCI was 2.0% and of naMCI 3.9% (see Table 3). Prevalence estimates of aMCI did not differ significantly across age groups or by sex (S1 Fig). Prevalence estimates of naMCI were greater among 70–79 year-olds than 60–69 year-olds for men (χ^2^ = 5.06, df = 1, *P* = .024), and greater among 80–89 year-olds than 70–79 year-olds for women (χ^2^ = 4.95, df = 1, *P* = .026), but there were no sex differences. On the basis of standardized estimates, there were no differences in the prevalence of either subtype between Whites (2.1%, 1.8–2.4% for aMCI and 3.8%, 3.4–4.2% for naMCI) and Chinese (1.8%, 1.1–2.5% for aMCI and 3.6%, 2.6–4.6% for naMCI; χ^2^ = 0.19, df = 1, *P* = .663 and χ^2^ = 0.39, df = 1, *P* = .532, respectively).

### Education and MCI

Compared to the lowest level (not having completed high school), all higher levels of education conveyed a reduced likelihood of MCI. The odds ratios were 0.58 (*P* < .001) for having completed high school, 0.55 (*P* < .001) for technical college or a diploma, and 0.73 (*P* = .002) for a university degree (S18 Table). Controlling for education however only partially reduced variation in prevalence across studies (further described in S2 Text).

### Effects of age on subjective memory complaints, functional independence, and objective cognitive impairment

We conducted supplementary analyses to determine the extent to which effects of age on the harmonized variables used to classify MCI may have contributed to our finding that the prevalence of MCI increased with age (values are detailed in S1–S3 Tables). The rate of subjective memory complaints increased with age, from 26.4% among 60–69 year-olds to 30.7% among 70–79 year-olds (χ^2^ = 33.95, df = 1, *P* < .001), and to 37.5% among 80–89 year-olds (χ^2^ = 67.58, df = 1, *P* < .001 vs. 70–79 year-olds). Conversely, the rates of functional independence decreased with age, from 97.7% among 60–69 year-olds to 94.5% among 70–79 year-olds (χ^2^ = 112.63, df = 1, *P* < .001), and to 83.8% among 80–89 year-olds (χ^2^ = 480.77, df = 1, *P* < .001 vs. 70–79 year-olds). There was no significant difference in the rate of objective cognitive impairment between 60–69 year-olds (20.6%) and 70–79 year-olds (19.5%; χ^2^ = 1.84, df = 1, *P* = .175), though the rate was higher for 80–89 year-olds (24.5%) than for 70–79 year-olds (χ^2^ = 30.06, df = 1, *P* < .001).

## Discussion

We analysed pooled data from 11 international cohort studies and found that applying uniform criteria to harmonized data greatly reduced the variation in MCI prevalence internationally. This was the case with each of the three definitions of cognitive impairment that we used to make separate classifications of MCI: performance in the bottom 6.681%, CDR of 0.5, and MMSE score of 24–27. The overall estimates found with these methods were between 6% and 12%, at the lower end of the 3% to 42% range reported by the international studies included in a recent review [14]. Our estimates for the studies contributing to the present investigation are also mostly lower than they themselves have previously published (Fig 1), which is due to the differential criteria used. Differences in the criteria for objective cognitive impairment may be particularly relevant [20], even in some cases where the same −1.5 SD threshold recommended by published criteria [11, 54] is used (and as per our definition of impairment being performance in the bottom 6.681%). For example, the criterion for impairment in the Sydney MAS was performance on any one of 12 individual neuropsychological tests being 1.5 SDs below published normative values [51]. In contrast, the COSMIC criterion for impairment was one of only four cognitive domain scores <−1.5 SD. The COSMIC approach of applying performance thresholds to domain scores, rather than to individual tests, was taken to prevent different prevalence estimates of MCI resulting merely from differences in the number of tests administered. The use of the −1.5 SD threshold in COSMIC also made a difference; e.g., EAS had previously used a 20^th^ percentile (approximately −0.84 SD) threshold [16]. For purposes of harmonization, we used only the most generalized self-report item from each study to ascertain the presence of a cognitive complaint. However, some of the contributing studies administered multiple cognitive complaint questions and/or scales to their participants, and sometimes also sought informant reports. With multiple opportunities, a participant may be more likely to endorse a complaint and thus more likely to be classified as having MCI (if meeting the other criteria). This could help account for why the prevalences of MCI we determined are lower than those previously reported by some studies. It must also be stated that the exclusion of dementia for this analysis altered the denominator in some of the studies, thereby influencing the prevalence estimates, albeit to a small extent only. Note that not all studies contained participants with dementia in their original samples, with some excluding individuals with dementia at the time of recruitment.

There being higher prevalence of MCI when less stringent criteria are applied reinforces the need for a harmonized approach. Compared to the −1.5 SD threshold for cognitive impairment we used, the DSM-5 definition of mild neurocognitive disorder recommends a less stringent criterion of −1.0 to −2.0 SD, without specifying how many tests should comprise a domain. Of course, the use of a threshold assumes that normative data are available for each test. We derived norms from the non-demented population of the same study. This approach offers the advantage of removing potential sociocultural and ethnic biases in any external source of normative data uniformly applied across all studies, and a similar approach was used by the 10/66 group [15]. A disadvantage is that it will mask the true extent of real differences in the populations themselves. In our study, use of this approach produced nearly identical percentages of impairment within each cognitive domain investigated. However, there were overall differences in impairment between studies because of differences in the number of individuals with impairment in multiple domains (see S3 Table). Even so, differences in MCI criteria other than objective cognitive impairment, including subjective memory complaints and functional independence, should be considered as major influences on the differences in MCI prevalence estimates we observed when using this particular approach.

Differences in MCI prevalence between studies may also stem from differences in methodology other than MCI criteria, including differences in recruitment procedures. It has been shown that including institutionalized individuals and obtaining proxy-based data for others either unable or unwilling to participate directly can elevate estimates of moderate and severe dementia prevalence [55]. A similar effect could be expected for MCI, and thus differences in response rates and the inclusion of institutionalized individuals in 3 of the 11 samples are likely to have contributed to some of the pre-existing and remaining (post-harmonization) variance in MCI prevalence across the studies we investigated.

In our study, the prevalence was about twice as high if MMSE was used to define objective impairment. The MMSE has many limitations when used for this purpose: it has age and education biases [56]; it shows cultural and linguistic artefacts [57]; it is strongly influenced by verbal memory function while not covering all domains of cognition adequately; and, though adopted as a definition for MCI by others [41, 42], the range of 24–27 for MCI has not been validated. Prevalence estimates using a CDR score of 0.5 as the basis for cognitive impairment were in between the estimates based on MMSE scores and those based on harmonized cognitive domain scores. A CDR score of 0.5 is a commonly-used criteria for MCI (e.g., [45, 46]). However, the use of CDR for the diagnosis of MCI has not been established; it is informant-dependent and requires clinical judgment that is difficult to standardize, and its correspondence with the generally accepted criteria for MCI is low in our study.

We did not find any significant sex differences in MCI prevalence. This is consistent with some previous literature [7, 15], but a possibly higher prevalence in men has also been reported [18]. We did find that the prevalence of MCI increased with age, though the pattern across age groups differed between men and women. There were also differences in how the prevalence of MCI increased with age across the three definitions of cognitive impairment we used. Prevalences determined on the basis of MMSE scores exhibited the largest increase with age, whereas those determined on the basis of performance in the bottom 6.681% showed the smallest (see Fig 2). The smaller changes observed with the latter definition are not surprising, given that age effects were likely underestimated by the use of age-adjusted neuropsychological test scores. Supplementary analyses on the harmonized variables used in classifying MCI suggest that an increase across the age groups in subjective memory complaints helped drive the increase in MCI with age. Conversely, rates of functional independence decreased with age. The increase in prevalence with age we found for MCI based on MMSE scores comes closest (of the different definitions used) to matching the exponential rise with age found for the global prevalence of dementia [58]. That study also found the global prevalence of dementia to be greater among women than among men, which differs from our findings for MCI. This suggests that MCI should not be conceptualized solely as a pre-dementia syndrome, but as a distinct syndrome with some overlap with pre-dementia. This, and MCI being associated with subtle impairments in high level or complex functional tasks [11, 13] that pose a burden on the individual and society, makes the prevalence of MCI of great interest in its own right.

We observed higher rates of MCI for White European populations than for Chinese when these were based on MMSE scores. Direct comparisons of dementia rates between Chinese and Whites appear to be scarce, and even more so for MCI. Previous work suggests the possibility of lower rates of dementia in China and Singapore compared to rates for the USA and Europe [59]. While loosely consistent with this, our findings of higher rates of MCI among Whites should be viewed very tentatively for a number of reasons. These include limitations in using the MMSE to classify MCI (as described above), there being no difference in rates between Chinese and Whites with the other definitions for MCI, and neither of the Chinese samples we investigated coming from mainland China.

The prevalence of MCI was lower in those with higher education, despite the neuropsychological test results having been corrected for education. Compared to not having completed high school, an apparent protective effect was found for all levels of education from having completed high school and above. However, though this effect was significant for having tertiary education (odds ratio = 0.73), it was not as strong as for either having completed high school (odds ratio = 0.58) or technical college/diploma (odds ratio = 0.55). The likely protective effect of education is consistent with the literature of a lower prevalence of dementia [60] and cognitive decline [61] in those with higher education.

It is noteworthy that our analysis showed higher prevalence estimates of naMCI to aMCI by a ratio of nearly 2:1, which is consistent with some studies [62, 63] but contrary to another [7]. The literature, however, has not examined naMCI with the same rigor as aMCI, and the common use of the 1999 Mayo Clinic criteria for MCI [54] conflates aMCI with MCI. It is arguable that our finding is artefactual since there are three non-memory domains, and it is thus statistically more likely for an individual to have impairment in a non-memory domain than in the sole memory domain. However, it may also be the case that a higher prevalence of naMCI stems from this being a more heterogeneous and unstable condition than aMCI, as evidenced by less progression to dementia and higher rates of reversion to normal cognition upon follow-up [64–66]. This issue deserves further study as it has potentially important clinical implications.

Strengths of our study include the large number of independent cohorts examined from diverse geographical, ethnic and sociocultural groups, and rigorous standardization of data. However, there are also some weaknesses. While the samples were epidemiologically derived, they were from specific regions and cannot be said to be necessarily representative of the countries or entire populations they come from. While all studies are longitudinal, this analysis is cross-sectional. Different instruments were used in the different studies, and ideally the harmonization process should begin at the time of study inception and not retrospectively. Cultural effects are also not to be ignored. For example, differences in rates of functional independence between studies could be influenced by cultural differences in reporting disability [67]. This in turn could contribute to differences in the estimated prevalences of MCI between studies (though the study with the lowest rate of dependence, ESPRIT, also had a lower prevalence of MCI than the study with the highest rate of dependence, WHICAP). Two studies had Chinese populations, but both were in high income countries, and while the American studies had some participants from black and Hispanic communities, these data are not presented separately. We have also restricted the analyses to prevalence figures and not examined determinants, which is worthy of future work.

## Supporting Information

S1 Fig Overall crude prevalence estimates of both amnestic mild cognitive impairment (aMCI) and non-amnestic mild cognitive impairment (naMCI) among men and women of different age groups. (DOCX)

S1 Table Prevalence estimates of subjective memory complaints or concerns. (DOCX)

S2 Table Prevalence estimates of functional independence. (DOCX)

S3 Table Prevalence estimates of objective cognitive impairment based on harmonized cognitive domain scores. (DOCX)

S4 Table Prevalence estimates of objective cognitive impairment based on Mini-Mental State Examination scores. (DOCX)

S5 Table Prevalence estimates of objective cognitive impairment based on Clinical Dementia Ratings. (DOCX)

S6 Table Recoding of original education data into COSMIC categories. (DOCX)

S7 Table Sources of Instrumental Activities of Daily Living items used. (DOCX)

S8 Table Coding of responses for harmonized Telephone, Food preparation, and Medications items. (DOCX)

S9 Table Coding of responses for harmonized Shopping, Finances, and Transport items. (DOCX)

S10 Table Tests or test components assigned to the memory domain. (DOCX)

S11 Table Tests or test components assigned to the attention/processing speed domain. (DOCX)

S12 Table Tests or test components assigned to the language domain. (DOCX)

S13 Table Tests or test components assigned to the executive function domain. (DOCX)

S14 Table Tests or test components assigned to the perceptual-motor domain. (DOCX)

S15 Table Memory complaint questions. (DOCX)

S16 Table Dementia criteria and Clinical Dementia Rating use. (DOCX)

S17 Table Sample sizes for the different approaches to classifying mild cognitive impairment. (DOCX)

S18 Table Association between education and mild cognitive impairment. (DOCX)

S1 Text Supplementary Methods. (DOCX)

S2 Text Supplementary Results. (DOCX)
