# Supplementary material for: The Prevalence of Mild Cognitive Impairment in Diverse Geographical and Ethnocultural Regions: The COSMIC Collaboration
Source: PLoS One. 2015 Nov 5;10(11):e0142388. doi: 10.1371/journal.pone.0142388 (PMC4634954; doi:10.1371/journal.pone.0142388)
Supplement: S1 Fig — (DOCX) [file pone.0142388.s001.docx]

## S1 Fig. Overall crude prevalence estimates of both amnestic mild cognitive impairment (aMCI) and non-amnestic mild cognitive impairment (naMCI) among men and women of different age groups. Error bars indicate upper limits of 95% confidence intervals. Asterisks indicate a significant difference from: * 60-69 years; ** 70-79 years.
