# Supplementary material for: The Prevalence of Mild Cognitive Impairment in Diverse Geographical and Ethnocultural Regions: The COSMIC Collaboration
Source: PLoS One. 2015 Nov 5;10(11):e0142388. doi: 10.1371/journal.pone.0142388 (PMC4634954; doi:10.1371/journal.pone.0142388)
Supplement: S1 Table — (DOCX) [file pone.0142388.s002.docx]

## S1 Table. Prevalence estimates of subjective memory complaints or concerns.

|  | **CFAS^a^** | **EAS** | **ESPRIT** | **HK-MAPS** | **Invece.Ab** | **MoVIES** | **PATH** | **SLAS** | **Sydney MAS** | **WHICAP** | **ZARADEMP** | **Total** |
| --- | --- | --- | --- | --- | --- | --- | --- | --- | --- | --- | --- | --- |
| Full sample | 22·6  535/2031 | 15·2  278/1824 | 19·6  420/2139 | 26·3  205/779 | 39·1  484/1239 | 36·6  467/1276 | 18·3  305/1669 | 18·0  426/2362 | 64·9  617/951 | 32·5  1276/3930 | 45·1  1896/4205 | 30·8  6909/22405 |
| Men | 23·7  201/759 | 15·4  110/714 | 19·8  177/892 | 24·2  87/360 | 38·2  218/570 | 41·5  208/501 | 22·7  192/846 | 15·4  143/928 | 64·2  280/436 | 27·5  353/1285 | 39·0  703/1802 | 29·4  2672/9093 |
| Women | 21·7 334/1272 | 15·1  168/1110 | 19·5  243/1247 | 28·2  118/419 | 39·8  266/669 | 33·4  259/775 | 13·7  113/823 | 19·7  283/1434 | 65·4  337/515 | 34·9  923/2645 | 49·6  1193/2403 | 31·8  4237/13312 |
| Age 60–69 | 18·6  113/480 | 0·0  0/20 | 16·7  108/645 | 30·0  88/293 | NA | 29·7  85/286 | 17·2  81/470 | 17·5  249/1424 | NA | 27·7  162/584 | 39·7  669/1684 | 26·4  1555/5886 |
| Age 70–79 | 22·3  238/942 | 14·4  159/1108 | 19·8  239/1206 | 25·6  92/360 | 39·1  484/1239 | 37·1  285/769 | 18·7  224/1199 | 19·0  145/763 | 66·0  374/567 | 31·8  680/2137 | 48·0  650/1355 | 30·7  3570/11645 |
| Age 80–89 | 29·0  162/528 | 17·1  111/651 | 25·4  68/268 | 20·0  23/115 | NA | 45·4  94/207 | NA | 18·0  30/167 | 63·4  239/377 | 37·5  404/1078 | 52·1  502/964 | 37·5  1633/4355 |

NA = not applicable. Values are presented as percentage and no./N, with no. = number of individuals classified as having a subjective memory complaint or concern and N = total number of individuals from the contributed sample with complete data for the subjective memory complaints or concerns variable. The numbers for the Full sample, Men and Women rows includes participants aged 90 years or more (and thus may not match the sum of numbers for the Age 60–69, Age 70–79 and Age 80–89 rows).

**^a^** CFAS percentages are weighted for study design.
