# Supplementary material for: The Prevalence of Mild Cognitive Impairment in Diverse Geographical and Ethnocultural Regions: The COSMIC Collaboration
Source: PLoS One. 2015 Nov 5;10(11):e0142388. doi: 10.1371/journal.pone.0142388 (PMC4634954; doi:10.1371/journal.pone.0142388)
Supplement: S3 Table — (DOCX) [file pone.0142388.s004.docx]

## S3 Table. Prevalence estimates of objective cognitive impairment based on harmonized cognitive domain scores.

| **Any domain** | | | | | | | | | | |
| --- | --- | --- | --- | --- | --- | --- | --- | --- | --- | --- |
|  | **EAS** | **ESPRIT** | **HK-MAPS** | **Invece.Ab** | **MoVIES** | **PATH** | **SLAS** | **Sydney MAS** | **WHICAP** | **Total** |
| Full sample | 20·5  386/1884 | 20·8  431/2068 | 24·0  104/433 | 17·9  225/1232 | 18·8  236/1252 | 19·3  366/1899 | 21·5  377/1756 | 19·1  196/1024 | 23·5  751/3198 | 20·8  3072/14746 |
| Men | 22·8  168/738 | 21·6  184/852 | 21·8  45/206 | 17·4  98/563 | 19·3  95/493 | 17·6  173/981 | 22·4  154/687 | 19·8  91/460 | 22·0  233/1059 | 20·5  1241/6039 |
| Women | 19·0  218/1146 | 20·3  247/1216 | 26·0  59/227 | 19·0  127/669 | 18·6  141/759 | 21·0  193/918 | 20·9  223/1069 | 18·6  105/564 | 24·2  518/2139 | 21·0  1831/8707 |
| Age 60–69 | 42·9  9/21 | 21·7  138/637 | 14·9  44/183 | NA | 17·4  49/282 | 19·7  106/537 | 21·1  233/1102 | NA | 18·8  90/480 | 20·6  669/3242 |
| Age 70–79 | 19·3  222/1151 | 18·8  217/1156 | 24·3  49/202 | 17·9  225/1232 | 15·5  118/759 | 19·1  260/1362 | 23·2  129/555 | 18·7  116/621 | 21·7  384/1771 | 19·5  1720/8809 |
| Age 80–89 | 21·3  142/666 | 26·5  68/257 | 23·4  11/47 | NA | 33·0  65/197 | NA | 15·1  14/93 | 19·6  78/397 | 27·9  239/857 | 24·5  617/2514 |
| **Memory domain** | | | | | | | | | | |
|  | **EAS** | **ESPRIT** | **HK-MAPS** | **Invece·Ab** | **MoVIES** | **PATH** | **SLAS** | **Sydney MAS** | **WHICAP** | **Total** |
| Full sample | 6·6  126/1899 | 6·6  144/2167 | 6·5  26/401 | 6·7  82/1232 | 6·6  84/1264 | 6·7  129/1937 | 6·6  117/1765 | 6·7  69/1037 | 6·7  265/3981 | 6·6  1042/15683 |
| Men | 7·1  53/743 | 7·4  67/901 | 7·8  15/192 | 6·2  35/563 | 5·2  26/500 | 6·1  61/1000 | 7·5  52/693 | 6·9  32/465 | 6·3  82/1305 | 6·6  423/6362 |
| Women | 6·3  73/1156 | 6·1  77/1266 | 5·3  11/209 | 7·0  47/669 | 7·6  58/764 | 7·3  68/937 | 6·1  65/1072 | 6·5  37/572 | 6·8  183/2676 | 6·6  619/9321 |
| Age 60–69 | 9·5  2/21 | 8·1  53/654 | 9·6  17/178 | NA | 5·3  15/284 | 7·1  39/547 | 6·2  69/1109 | NA | 6·1  36/589 | 6·8  231/3382 |
| Age 70–79 | 5·1  59/1162 | 5·6  68/1222 | 3·4  6/179 | 6·7  82/1232 | 6·7  51/765 | 6·5  90/1390 | 8·1  45/558 | 6·4  40/626 | 6·5  141/2169 | 6·3  582/9303 |
| Age 80–89 | 8·8  59/670 | 7·7  21/272 | 7·0  3/43 | NA | 8·5  17/201 | NA | 3·2  3/93 | 6·9  28/404 | 7·6  83/1089 | 7·7  214/2772 |
| **Non–memory domain** | | | | | | | | | | |
|  | **EAS** | **ESPRIT** | **HK-MAPS** | **Invece·Ab** | **MoVIES** | **PATH** | **SLAS** | **Sydney MAS** | **WHICAP** | **Total** |
| Full sample | 13·7  257/1875 | 13·2  267/2030 | 13·6  56/411 | 12·4  143/1150 | 12·0  150/1246 | 12·2  230/1881 | 12·6  215/1700 | 12·3  126/1021 | 12·3  363/2956 | 12·7  1807/14270 |
| Men | 15·6  115/736 | 13·4  112/834 | 10·2  20/196 | 11·9  63/528 | 14·1  69/491 | 11·1  108/973 | 12·1  80/661 | 12·7  58/458 | 11·7  116/993 | 12·6  741/5870 |
| Women | 12·5  142/1139 | 13·0  155/1196 | 16·7  36/215 | 12·9  80/622 | 10·7  81/755 | 13·4  122/908 | 13·0  135/1039 | 12·1  68/563 | 12·6  247/1963 | 12·7  1066/8400 |
| Age 60–69 | 33·3  7/21 | 12·8  80/627 | 11·9  21/177 | NA | 12·1  34/281 | 12·5  67/536 | 12·9  138/1067 | NA | 9·3  42/453 | 12·3  389/3162 |
| Age 70–79 | 14·1  162/1150 | 12·3  139/1134 | 16·3  31/190 | 12·4  143/1150 | 8·9  67/757 | 12·1  163/1345 | 12·8  69/539 | 12·1  75/619 | 11·4  189/1652 | 12·2  1038/8536 |
| Age 80–89 | 12·3  81/658 | 17·1  43/252 | 9·3  4/43 | NA | 23·7  46/194 | NA | 9·0  8/89 | 12·6  50/396 | 14·4  112/777 | 14·3  344/2409 |

NA = not applicable. Values are presented as percentage and no./N, with no. = number of individuals with a relevant cognitive domain score <−1.5 SD and N = total number of individuals from the contributed sample with sufficient cognitive domain data. Numbers for memory and non-memory domain estimates do not sum to corresponding estimates for any domain because non-impaired individuals were excluded only from calculations for which they had insufficient data (e.g., they may be counted for the memory domain but not counted for the non-memory domain). Individuals were also excluded from the non-memory domain estimate calculations if having a memory domain impairment or missing memory domain data. The numbers for the Full sample, Men and Women rows includes participants aged 90 years or more (and thus may not match the sum of numbers for the Age 60–69, Age 70–79 and Age 80–89 rows).
