# Supplementary material for: The Prevalence of Mild Cognitive Impairment in Diverse Geographical and Ethnocultural Regions: The COSMIC Collaboration
Source: PLoS One. 2015 Nov 5;10(11):e0142388. doi: 10.1371/journal.pone.0142388 (PMC4634954; doi:10.1371/journal.pone.0142388)
Supplement: S5 Table — (DOCX) [file pone.0142388.s006.docx]

## S5 Table. Prevalence estimates of objective cognitive impairment based on Clinical Dementia Ratings.

|  | **EAS** | **HK-MAPS** | **MoVIES** | **SLAS** | **Sydney MAS** | **WHICAP** | **Total** |
| --- | --- | --- | --- | --- | --- | --- | --- |
| Full sample | 27·2  414/1523 | 51·4  404/786 | 3·9  50/1276 | 50·1  376/751 | 14·6  137/939 | 25·2  1005/3982 | 28·5  3166/11109 |
| Men | 27·5  164/597 | 47·3  172/364 | 5·2  26/501 | 49·3  144/292 | 18·2  75/412 | 25·2  328/1303 | 28·6  1234/4311 |
| Women | 27·0  250/926 | 55·0  232/422 | 3·1  24/775 | 50·5  232/459 | 11·8  62/527 | 25·3  677/2679 | 28·4  1932/6798 |
| Age 60–69 | 26·7  4/15 | 33·4  99/296 | 1·0  3/286 | 49·1  217/442 | NA | 14·7  86/584 | 27·5  688/2505 |
| Age 70–79 | 23·6  230/973 | 58·7  212/361 | 3·3  25/769 | 51·0  127/249 | 12·3  70/569 | 21·2  461/2172 | 25·2  1459/5791 |
| Age 80–89 | 32·9  164/499 | 70·3  83/118 | 10·1  21/207 | 50·0  28/56 | 18·4  67/365 | 35·8  391/1092 | 34·9  903/2585 |

NA = not applicable. Values are presented as percentage and no./N, with no. = number of individuals with a Clinical Dementia Rating of 0.5, and N = total number of individuals from the contributed sample with complete data for the Clinical Dementia Rating variable. The numbers for the Full sample, Men and Women rows includes participants aged 90 years or more (and thus may not match the sum of numbers for the Age 60–69, Age 70–79 and Age 80–89 rows).
