# Supplementary material for: The Prevalence of Mild Cognitive Impairment in Diverse Geographical and Ethnocultural Regions: The COSMIC Collaboration
Source: PLoS One. 2015 Nov 5;10(11):e0142388. doi: 10.1371/journal.pone.0142388 (PMC4634954; doi:10.1371/journal.pone.0142388)
Supplement: S6 Table — (DOCX) [file pone.0142388.s007.docx]

## S6 Table. Recoding of original education data into COSMIC categories.

| **Study** | **Original values used** | **Original value → COSMIC category** |
| --- | --- | --- |
| CFAS | Years | < 9 → 1  9-10 → 2  11-12 → 3  > 12 → 4 |
| EAS | 1. None 2. High school diploma/GED 3. Bachelors 4. Masters 5. Doctorate 6. Other | 1 → 1  2 → 2  3 → 4  4 → 4  5 → 4  6 → 3 |
| ESPRIT | 1. < 5th grade 2. 5th grade 3. 6th to 9th grade 4. Technical 9th grade 5. College 6. College graduate (including technical)   7. University | 1 → 1  2 → 1  3 → 2  4 → 2  5 → 2  6 → 3  7 → 4 |
| HK-MAPS | Years | < 12 → 1  12 → 2  13-14 → 3  > 14 → 4 |
| Invece.Ab | Years | < 13 → 1  13-16 → 2  > 16 → 4 |
| MoVIES | 1. Graduate/professional 2. College graduate 3. Partial college 4. Trade/technical 5. High school graduate 6. Partial high school 7. 6^th^-9^th^ grade 8. < 6^th^ grade | 1 → 4  2 → 3  3 → 2  4 → 3  5 → 2  6 → 1  7 → 1  8 → 1 |
| PATH | 1. Primary and secondary: 2. Some primary 3. All of primary 4. Some secondary 5. Three/four years of secondary (intermediate certificate) 6. Five/six years of secondary (higher school certificate) 7. Post-secondary schooling: 8. Trade certificate/apprenticeship 9. Technicians certificate/advanced certificate 10. Certificate other than above 11. Associate diploma 12. Undergraduate diploma 13. Bachelors degree 14. Postgraduate diploma/certificate 15. Higher degree | 1 → 1  2 → 1  3 → 1  4 → 1  5 → 2  1 → 1 if A = 1-4  1 → 2 if A = 5  2 → 3  3 → 3  4 → 3  5 → 3  6 → 4  7 → 4  8 → 4 |
| SLASI/II | 1. None 2. Primary 3. Secondary or ITE 4. Pre-university or polytechnic 5. University   Years | 1 → 1  2 → 1  3 → 1 if Years < 12 or missing  3 → 2 if Years = 12  3 → 3 if Years > 12  4 → 3  5 → 4 |
| Sydney MAS | 1. Primary school 2. Incomplete high school 3. Completed high school 4. Incomplete tertiary 5. Completed tertiary 6. Incomplete high school + certificate/diploma 7. Completed high school + certificate/diploma | 1 → 1  2 → 1  3 → 2  4 → 2  5 → 4  6 → 3  7 → 3 |
| WHICAP | Years | < 12 → 1  12 → 2  13-15 → 3  > 15 → 4 |
| ZARADEMP | 1. None 2. Less than primary 3. Primary 4. Less than technical formation 5. Technical formation 6. Less than high school 7. High school 8. College diploma 9. University degree | 1 → 1  2 → 1  3 → 1  4 → 1  5 → 3  6 → 1  7 → 2  8 → 3  9 → 4 |

The COSMIC harmonized education variable values are:

1. Less than high school completion
2. High school completion
3. Technical or college diploma
4. University degree.

Each of the contributing studies helped determine how the data representing their local education system was best transformed to these categories.
