# Supplementary material for: The Prevalence of Mild Cognitive Impairment in Diverse Geographical and Ethnocultural Regions: The COSMIC Collaboration
Source: PLoS One. 2015 Nov 5;10(11):e0142388. doi: 10.1371/journal.pone.0142388 (PMC4634954; doi:10.1371/journal.pone.0142388)
Supplement: S7 Table — (DOCX) [file pone.0142388.s008.docx]

## S7 Table. Sources of Instrumental Activities of Daily Living items used.

| **Study** | **Instrument providing items** | **Notes** |
| --- | --- | --- |
| CFAS | Townsend Disability Scale | No harmonizable Finances, Medications or Telephone items |
| EAS | Lawton & Brody Scale | Men not administered the Food Preparation item |
| ESPRIT | Lawton & Brody Scale | Men not administered the Food Preparation item |
| HK-MAPS | Disability Assessment for Dementia Scale (Chinese) |  |
| Invece.Ab | Lawton & Brody Scale |  |
| MoVIES | Older Adults Resources and Services (OARS) Scale |  |
| PATH | Items adapted from the US Health and Retirement Study | No harmonizable Finances item |
| SLASI/II | Lawton & Brody Scale | Data provided as recoded responses |
| Sydney MAS | Bayer ADL Scale |  |
| WHICAP | Disability and Functional Limitations Instrument | No harmonizable Transport item |
| ZARADEMP | Lawton & Brody Scale | Data provided as recoded responses |
