# Supplementary material for: The Prevalence of Mild Cognitive Impairment in Diverse Geographical and Ethnocultural Regions: The COSMIC Collaboration
Source: PLoS One. 2015 Nov 5;10(11):e0142388. doi: 10.1371/journal.pone.0142388 (PMC4634954; doi:10.1371/journal.pone.0142388)
Supplement: S8 Table — (DOCX) [file pone.0142388.s009.docx]

## S8 Table. Coding of responses for harmonized Telephone, Food preparation, and Medications items.

| **Study** | **Telephone** | **Food preparation** | **Medications** |
| --- | --- | --- | --- |
| CFAS | **-** | *Are you able to prepare and cook a hot meal? If YES: Do you have difficulty?*  1. No, needs help  2. Yes, some difficulty  **3. Yes, no difficulty**  *If hot meals are always prepared by someone else, could you prepare and cook a hot meal if you had to?*  1. No, needs help  2. Yes, some difficulty  **3. Yes, no difficulty** | **-** |
| EAS;  ESPRIT; Invece.Ab | 1. **Operates telephone on own initiative – looks up and dials numbers etc** 2. **Dials a few well-known numbers** 3. **Answers telephone but does not dial** 4. Does not use telephone at all | 1. **Plans, prepares and serves adequate meals independently** 2. Prepares adequate meals if supplied with ingredients 3. Heats and serves prepared meals, or prepares meals but does not maintain adequate diet 4. Needs to have meals prepared and served | 1. **Is responsible for taking medication in correct dosages at correct time** 2. Takes responsibility if medication is prepared in advance in separate dosages 3. Is not capable of dispensing own medication |
| HK-MAPS | 1. *Using the phone in a correct timing* 2. *Able to find out the correct number to dial, and correctly making a call* 3. *Able to take and deliver messages via the phone*   **Yes to any**  No to all | 1. *Initiate preparing snacks or meals when needed* 2. *Prepare simple snacks or meals* 3. *Cook*   **Yes to all**  No to any | 1. *Initiate the need to take medications at appropriate timing* 2. *Able to locate the medications and use correctly* 3. *Taking medications according to doctors' prescriptions*   **Yes to all**  No to any |
| MoVIES | *Can you use the telephone?*  **1. Without help**  2. With some help or completely unable | *Can you prepare your own meals?*  **1. Without help**  2. With some help or completely unable | *Can you take your own medicine?*  **1. Without help**  2. With some help or completely unable |
| PATH | *Do you have any difficulty making telephone calls?*   1. **No** 2. Yes 3. Can’t make telephone calls 4. Don’t make telephone calls | *Do you have any difficulty preparing a hot meal?*   1. **No** 2. Yes 3. Can’t prepare meals 4. Don’t prepare meals | *Do you have any difficulty taking medications?*   1. **No** 2. Yes 3. Can’t take medications   *If don’t take medication, do you think you would have any difficulty taking medication if you needed to do so?*   1. **No** 2. Yes 3. Don’t know |
| SLASI/II | 1. **Independent** 2. **Aided** 3. Dependent | 1. **1. Independent** 2. 2. Aided 3. 3. Dependent | 1. **1. Independent** 2. 2. Aided 3. 3. Dependent |
| Sydney MAS | *Difficulty using the telephone*  **1-4**  5-10 | *Difficulty with preparing food*  **1-2**  3-10 | *Difficulty with taking medication without supervision*  **1-2**  3-10 |
| WHICAP | *Used help dialling telephone no. in month*  **1. No**  2. Yes | *Preparing-cooking meals in month*   1. **All/most all by self** 2. Most by self/others do rest 3. None or almost none by self | *Help with medication*   1. **All meds by self** 2. Reminded 3. Someone give med |
| ZARADEMP | 1. **Independent** 2. **Need some help** 3. Dependent | 1. **1. Independent** 2. 2 Need some help 3. 3. Dependent | 1. **1. Independent** 2. 2. Need some help   3. Dependent |

Responses were coded as independent (bold font) or dependent (plain font). The shaded row details the Lawton & Brody Scale categories to which others were matched. Data for SLASI/II and ZARADEMP were provided by these studies as recoded from original Lawton & Brody Scale responses.
