# Supplementary material for: The Prevalence of Mild Cognitive Impairment in Diverse Geographical and Ethnocultural Regions: The COSMIC Collaboration
Source: PLoS One. 2015 Nov 5;10(11):e0142388. doi: 10.1371/journal.pone.0142388 (PMC4634954; doi:10.1371/journal.pone.0142388)
Supplement: S9 Table — (DOCX) [file pone.0142388.s010.docx]

## S9 Table. Coding of responses for harmonized Shopping, Finances, and Transport items.

| **Study** | **Shopping** | **Finances** | **Transport** |
| --- | --- | --- | --- |
| CFAS | *Are you able to shop and carry heavy bags? If YES: Do you have difficulty?*  1. No, needs help  **2. Yes, some difficulty**  **3. Yes, no difficulty** | **-** | *Are you able to get on a bus? If YES: Do you have difficulty?*  1. No, needs help  **2. Yes, some difficulty**  **3. Yes, no difficulty** |
| EAS;  ESPRIT; Invece.Ab | 1. **Takes care of all shopping needs independently** 2. Shops independently for small purchases 3. Needs to be accompanied on any shopping trip 4. Completely unable to shop | 1. **Manages financial matters independently (budgets, writes checks, pays rent, bills, goes to bank), collects and keeps track of income** 2. **Manages day-to-day purchases, but needs help with banking, major purchases, etc** 3. Incapable of handling money. | 1. **Travels independently on public transportation or drives own car** 2. **Arranges own travel via taxi, but does not otherwise use public transportation** 3. **Travels on public transportation when assisted or accompanied by another** 4. Travel limited to taxi or automobile with assistance of another |
| HK-MAPS | *Able to go out and buy appropriate things by self*  **Yes**  No | 1. *Managing own finance (e.g. paying bills, writing cheques, reading bank statements)* 2. *Doing transactions correctly (e.g. able to calculate the amount of changes when buying things)*   **Yes to either**  No to both | *Using public transport safely*  **Yes**  No |
| MoVIES | *Can you go shopping for groceries or clothes?*  **1. Without help**  2. With some help or completely unable | *Can you handle your own money?*  **1. Without help**  2. With some help or completely unable | *Can you get to places out of walking distance?*  **1. Without help**  2. With some help or completely unable |
| PATH | *Do you have any difficulty shopping for groceries?*   1. **No** 2. Yes 3. Can’t shop for groceries 4. Don’t shop for groceries | - | *Do you consider yourself a current driver?*  **Yes**  **-** |
| SLASI/SLASII | 1. **Independent** 2. Aided 3. Dependent | 1. **Independent** 2. **Aided** 3. Dependent | 1. **Independent** 2. **Aided** 3. Dependent |
| Sydney MAS | *Difficulty with shopping*  **1-2**  3-10 | *Difficulty understanding his/her personal financial affairs*  **1-4**  5-10 | *Difficulty using transportation*  **1-4**  5-10 |
| WHICAP | *Shopping in month*   1. **All/most all by self** 2. Most by self/others do rest 3. Some by self/others do rest 4. None or almost none by self | *Problems-difficulty handling personal business*  **No**  Yes | - |
| ZARADEMP | 1. **Independent** 2. Need some help 3. Dependent | 1. **Independent** 2. **Need some help** 3. Dependent | 1. **Independent** 2. **Need some help** 3. Dependent |

Responses were coded as independent (bold font) or dependent (plain font). The shaded row details the Lawton & Brody Scale categories to which others were matched. Data for SLASI/II and ZARADEMP were provided by these studies as recoded from original Lawton & Brody Scale responses.
