# Supplementary material for: The Prevalence of Mild Cognitive Impairment in Diverse Geographical and Ethnocultural Regions: The COSMIC Collaboration
Source: PLoS One. 2015 Nov 5;10(11):e0142388. doi: 10.1371/journal.pone.0142388 (PMC4634954; doi:10.1371/journal.pone.0142388)
Supplement: S10 Table — (DOCX) [file pone.0142388.s011.docx]

## S10 Table. Tests or test components assigned to the memory domain.

| **EAS** | **ESPRIT** | **HK-MAPS** | **Invece.Ab** | **MoVIES** | **PATH** | **SLAS I** | **SLAS II** | **Sydney MAS** | **WHICAP** |
| --- | --- | --- | --- | --- | --- | --- | --- | --- | --- |
| Story recall (WMS-R), Sum of A and B | Benton test | ADAS Cog word recall, average of trials 1-3 | RAVLT, Sum of trials 1-5 | Story recall (18-item), immediate recall | CVLT, immediate recall | RAVLT, sum of trials 1-5 | Story recall, local version A | RAVLT, sum of trials 1-5 | Selective reminding test, total recall |
| Free & cued selective reminding (Buschke) |  | ADAS Cog 10-min delayed recall of words in trials 1-3 | RAVLT, delayed recall (15 min) | Story recall (18-item), 30-min delayed recall | CVLT, delayed recall | RAVLT short term delayed recall | Story recall, local version B | RAVLT, trial 6 | Selective reminding test, delayed recall |
|  |  |  | Babcock story recall, sum of trials 1-2 | CERAD word list, immediate recall | Faces, correctly recognised | RAVLT long term delayed recall (30 min) | Story recall, local version A, delayed | RAVLT, trial 7 | Selective reminding test, delayed recognition |
|  |  |  | Rey-Osterrieth Complex Figure Recall | CERAD word list, delayed recall |  | Visual reproduction, delayed recall (WMS-III) | Story recall, local version B, delayed | Logical memory story A immediate recall (WMS-III) | Benton visual retention test, recognition |
|  |  |  |  |  |  |  | RAVLT, Sum of trials 1-5 | Logical memory story A delayed recall (WMS-III) |  |
|  |  |  |  |  |  |  | RAVLT short term delayed recall | Benton visual recognition test |  |
|  |  |  |  |  |  |  | RAVLT long term delayed recall (30 min) |  |  |
|  |  |  |  |  |  |  | Brief visuospatial memory test-R, delayed recall |  |  |
