# Supplementary material for: The Prevalence of Mild Cognitive Impairment in Diverse Geographical and Ethnocultural Regions: The COSMIC Collaboration
Source: PLoS One. 2015 Nov 5;10(11):e0142388. doi: 10.1371/journal.pone.0142388 (PMC4634954; doi:10.1371/journal.pone.0142388)
Supplement: S11 Table — (DOCX) [file pone.0142388.s012.docx]

## S11 Table. Tests or test components assigned to the attention/processing speed domain.

| **EAS** | **ESPRIT** | **HK-MAPS** | **Invece.Ab** | **MoVIES** | **PATH** | **SLAS I** | **SLAS II** | **Sydney MAS** | **WHICAP** |
| --- | --- | --- | --- | --- | --- | --- | --- | --- | --- |
| Digit span forward (WAIS-III/R) | Trail making test A | Trail making test A | Trail making test A | Trail making test A | Trail making test A | Trail making test A | Digit span forward (WAIS-III, extra trials) | Trail making test A | Color trails test |
| Digit symbol coding (WAIS-III/R) |  | Digit span forward (WAIS-R) | Attentional Matrices |  | Symbol digit modalities test, written | Symbol digits modalities test, written |  | Digit symbol coding (WAIS-III) |  |
|  |  | Visual memory span forward (WMS-R) |  |  |  | Symbol digits modalities test, oral |  |  |  |
|  |  |  |  |  |  | Digit span forward (WAIS-III, extra trials) |  |  |  |
|  |  |  |  |  |  | Spatial span forward |  |  |  |
