# Supplementary material for: The Prevalence of Mild Cognitive Impairment in Diverse Geographical and Ethnocultural Regions: The COSMIC Collaboration
Source: PLoS One. 2015 Nov 5;10(11):e0142388. doi: 10.1371/journal.pone.0142388 (PMC4634954; doi:10.1371/journal.pone.0142388)
Supplement: S12 Table — (DOCX) [file pone.0142388.s013.docx]

## S12 Table. Tests or test components assigned to the language domain.

| **EAS** | **ESPRIT** | **HK-MAPS** | **Invece.Ab** | **MoVIES** | **PATH** | **SLAS I** | **SLAS II** | **Sydney MAS** | **WHICAP** |
| --- | --- | --- | --- | --- | --- | --- | --- | --- | --- |
| Semantic fluency, total animals, fruits, vegetables | Isaac’s category fluency test: animals, colors, fruits and cities | Semantic fluency, animals | Semantic fluency, colors, animals, fruits and cities | Semantic fluency, fruits and animals | Boston naming test (15-item) | Semantic fluency, animals, fruits and vegetables | Semantic fluency, animals | Semantic fluency, animals | Semantic fluency, animals, food and clothing |
|  |  | Semantic fluency, fruits |  | Boston naming test (15-item) |  |  | Boston naming test (30-item) | Boston naming test (30-item) | Boston naming test (15-item) |
|  |  | Semantic fluency, vegetables |  |  |  |  |  |  |  |
