# Supplementary material for: The Prevalence of Mild Cognitive Impairment in Diverse Geographical and Ethnocultural Regions: The COSMIC Collaboration
Source: PLoS One. 2015 Nov 5;10(11):e0142388. doi: 10.1371/journal.pone.0142388 (PMC4634954; doi:10.1371/journal.pone.0142388)
Supplement: S13 Table — (DOCX) [file pone.0142388.s014.docx]

## S13 Table. Tests or test components assigned to the executive function domain.

| **EAS** | **ESPRIT** | **HK-MAPS** | **Invece.Ab** | **MoVIES** | **PATH** | **SLAS I** | **SLAS II** | **Sydney MAS** | **WHICAP** |
| --- | --- | --- | --- | --- | --- | --- | --- | --- | --- |
| Verbal fluency, total FAS | Trail making test B | Digit span backward (WAIS-R) | Phonemic verbal fluency | Trail making test B | Trail making test B | Trail making test B | Digit span backward (WAIS-III, extra trials) | Trail making test B | Similarities (WAIS-R) |
| Digit span backward (WAIS-III/R) |  | Visual memory span backward (WMS-R) | Trail making test B | Verbal fluency, PS | Verbal fluency, F | Digit span backward (WAIS-III, extra trials) |  | Verbal fluency, FAS | Identities and similarities (Mattis dementia rating scale) |
|  |  | Trail making test B | Raven’s Coloured Matrices |  | Verbal fluency, A | Design fluency (D-KEFS) |  |  | Verbal fluency, CFL (PSV for Spanish speakers) |
|  |  |  |  |  | Digit span backward (WAIS-III truncated) | Spatial span backward (WMS-III) |  |  |  |
