# Supplementary material for: The Prevalence of Mild Cognitive Impairment in Diverse Geographical and Ethnocultural Regions: The COSMIC Collaboration
Source: PLoS One. 2015 Nov 5;10(11):e0142388. doi: 10.1371/journal.pone.0142388 (PMC4634954; doi:10.1371/journal.pone.0142388)
Supplement: S14 Table — (DOCX) [file pone.0142388.s015.docx]

## S14 Table. Tests or test components assigned to the perceptual-motor domain.

| **EAS** | **MoVIES** | **SLAS I** | **SLAS II** | **Sydney MAS** | **WHICAP** |
| --- | --- | --- | --- | --- | --- |
| Block design (WAIS-III/R) | Clock drawing | Block design (WAIS-III) | Block design (WAIS-III) | Block design (WAIS-R) | Rosen drawing test, 5 construction items |
|  | CERAD constructional praxis |  |  |  | Benton visual retention test, matching (10 items, form C) |
