# Supplementary material for: The Prevalence of Mild Cognitive Impairment in Diverse Geographical and Ethnocultural Regions: The COSMIC Collaboration
Source: PLoS One. 2015 Nov 5;10(11):e0142388. doi: 10.1371/journal.pone.0142388 (PMC4634954; doi:10.1371/journal.pone.0142388)
Supplement: S15 Table — (DOCX) [file pone.0142388.s016.docx]

## S15 Table. Memory complaint questions.

| **Study** | **Question** | **Response options** |
| --- | --- | --- |
| CFAS | Have you ever had any difficulty with your memory? | **Yes,** No |
| EAS | Difficulty remembering things that happened recently? | **Yes,** No |
| ESPRIT | Do you forget everyday things? *Over the past year* | **Yes,** No |
| HK-MAPS | Have you been concerned about your memory? *In the past month* | **Yes,** No |
| Invece.Ab | Do you have any problems with your memory? | **Yes,** No |
| MoVIES | In general, how good do you feel your memory is for a person your age? | **Poor, Fair**,  Good, Excellent |
| PATH | Remembering things that have happened recently *compared to 4 years ago* | **Much worse, A bit worse,**  Not much change, A bit improved, Much better |
| SLASI | Do you have problems with memory or thinking? | **Yes,** No |
| SLASII | Overall, how would you rate your memory or other mental abilities as compared to others of similar age? | **Much worse, A bit worse,**  Not much different, A bit better, Much better |
| Sydney MAS | Have you noticed difficulties with your memory? | **Yes,** No |
| WHICAP | Do you have any problems with your memory? | **Yes,** No |
| ZARADEMP | Have you had any difficulty with your memory? | **Yes,** No |

Response options in bold font indicate a complaint.
