# Supplementary material for: The Prevalence of Mild Cognitive Impairment in Diverse Geographical and Ethnocultural Regions: The COSMIC Collaboration
Source: PLoS One. 2015 Nov 5;10(11):e0142388. doi: 10.1371/journal.pone.0142388 (PMC4634954; doi:10.1371/journal.pone.0142388)
Supplement: S16 Table — (DOCX) [file pone.0142388.s017.docx]

## S16 Table. Dementia criteria and Clinical Dementia Rating use.

| **Study** | **Dementia criteria** | **CDR** |
| --- | --- | --- |
| CFAS | AGE-CAT | No |
| EAS | DSM-IV | Yes |
| ESPRIT | Neurological^a^ | No |
| HK-MAPS | CDR ≥ 1 | Yes |
| Invece.Ab | DSM-IV | No |
| MoVIES | CDR ≥ 1 | Yes |
| PATH | DSM-IV | Yes |
| SLASI/SLASII | DSM-IV | Yes |
| Sydney MAS | DSM-IV | Yes |
| WHICAP | DSM-IV | Yes |
| ZARADEMP | DSM-IV | No |

CDR = Clinical Dementia Rating.

^a^ Standardized interview by a neurologist incorporating cognitive testing, with diagnoses validated by an independent panel of expert neurologists.
