# Supplementary material for: The Prevalence of Mild Cognitive Impairment in Diverse Geographical and Ethnocultural Regions: The COSMIC Collaboration
Source: PLoS One. 2015 Nov 5;10(11):e0142388. doi: 10.1371/journal.pone.0142388 (PMC4634954; doi:10.1371/journal.pone.0142388)
Supplement: S17 Table — (DOCX) [file pone.0142388.s018.docx]

## S17 Table. Sample sizes for the different approaches to classifying mild cognitive impairment.

| **Study** | **MCI** | **aMCI** | **naMCI** | **MMSE** | **CDR** |
| --- | --- | --- | --- | --- | --- |
| CFAS | NA | NA | NA | 987 | NA |
| EAS | 1782 | 1780 | 1796 | 1580 | 1438 |
| ESPRIT | 2017 | 1982 | 2114 | 2012 | NA |
| HK-MAPS | 427 | 427 | 396 | 627 | 760 |
| Invece.Ab | 1177 | 1091 | 1172 | 1103 | NA |
| MoVIES | 1252 | 1246 | 1264 | 1194 | 1276 |
| PATH | 1603 | 1590 | 1642 | 1653 | NA |
| SLAS | 1410 | 1383 | 1393 | 2168 | 742 |
| Sydney MAS | 881 | 878 | 892 | 884 | 848 |
| WHICAP | 3067 | 2876 | 3748 | NA | 3748 |
| ZARADEMP | NA | NA | NA | 3381 | NA |

NA = not applicable.
