# Supplementary material for: The Prevalence of Mild Cognitive Impairment in Diverse Geographical and Ethnocultural Regions: The COSMIC Collaboration
Source: PLoS One. 2015 Nov 5;10(11):e0142388. doi: 10.1371/journal.pone.0142388 (PMC4634954; doi:10.1371/journal.pone.0142388)
Supplement: S18 Table — (DOCX) [file pone.0142388.s019.docx]

## S18 Table. Association between education and mild cognitive impairment.

| **Study** | **Education** | | **Completed high school** | | **Technical college/diploma** | | **University degree** | | **Age** | | | **Sex** | |
| --- | --- | --- | --- | --- | --- | --- | --- | --- | --- | --- | --- | --- | --- |
|  | **Wald** | ***P*** | **OR** | ***P*** | **OR** | ***P*** | **OR** | ***P*** | | **OR** | ***P*** | **OR** | ***P*** |
| EAS | 6·447 | 0·092 | 0·525 | 0·029 | 0·739 | 0·587 | 0·972 | 0·925 | | 0·986 | 0·519 | 0·674 | 0·088 |
| ESPRIT | 13·997 | 0·003 | 0·600 | 0·031 | 0·195 | 0·007 | 0·413 | 0·004 | | 1·023 | 0·205 | 0·952 | 0·821 |
| HK-MAPS | 0·235 | 0·972 | 0·667 | 0·699 | 0·000 | 0·999 | 0·717 | 0·753 | | 1·046 | 0·155 | 1·135 | 0·773 |
| Invece.Ab | 4·771 | 0·092 | 0·275 | 0·077 | - | - | 1·966 | 0·227 | | 0·985 | 0·859 | 1·029 | 0·898 |
| MoVIES | 6·304 | 0·098 | 0·543 | 0·016 | 0·694 | 0·263 | 0·505 | 0·358 | | 1·020 | 0·321 | 0·797 | 0·305 |
| PATH | 3·161 | 0·367 | 0·598 | 0·432 | 0·812 | 0·609 | 1·353 | 0·406 | | 0·981 | 0·840 | 0·957 | 0·879 |
| SLAS | 0·391 | 0·942 | 0·975 | 0·973 | 0·687 | 0·535 | 1·037 | 0·961 | | 0·991 | 0·649 | 1·112 | 0·681 |
| Sydney MAS | 0·125 | 0·989 | 0·899 | 0·740 | 0·987 | 0·971 | 1·000 | 0·999 | | 0·995 | 0·840 | 1·168 | 0·486 |
| WHICAP | 12·987 | 0·005 | 0·565 | 0·008 | 0·516 | 0·013 | 0·655 | 0·051 | | 0·998 | 0·842 | 1·391 | 0·047 |
| Pooled sample | 44·951 | <0·001 | 0·579 | <0·001 | 0·546 | <0·001 | 0·732 | 0·002 | | 1·018 | 0·002 | 1·035 | 0·648 |

OR = odds ratio. Results of logistic regressions for each study and the pooled sample of studies contributing sufficient raw data, with mild cognitive impairment (yes = 1, no = 0) as the dependent variable and education, age and sex (female = 1, male = 0) as independent variables. The reference category against which each of the three education levels is compared is “Less than completed high school”.
