# Supplementary material for: The Prevalence of Mild Cognitive Impairment in Diverse Geographical and Ethnocultural Regions: The COSMIC Collaboration
Source: PLoS One. 2015 Nov 5;10(11):e0142388. doi: 10.1371/journal.pone.0142388 (PMC4634954; doi:10.1371/journal.pone.0142388)
Supplement: S1 Text — (DOCX) [file pone.0142388.s020.docx]

## S1 Text. Supplementary Methods

## *Selection of neuropsychological tests to represent cognitive domains*

Inspection of the range of tests available from each study led to the decision to attempt to obtain domain scores from as many studies as possible for the following five domains: memory, attention/processing speed (PS), language, executive function, and perceptual-motor. Tests were allocated to domains to be consistent with common practice [[1-3](#_ENREF_1)], although it is acknowledged that this does vary somewhat, and might not always be consistent with the way that the individual participating studies might have formed domains. However, an attempt was made to maintain the same general principles used across the studies.

Some of the general principles adopted that might vary from those used by other studies include:

- Tests such as backward digit span were regarded as executive function measures, rather than memory, or attention. This is consistent with a psychometric study that allocated backward digit span to an executive function scale on the basis of item response theory [[4](#_ENREF_4)].
- Digit Span Forward and other similar tests were treated as measures of attention/processing speed (PS), rather than of memory. Digit Span Forward is considered a measure of elementary attention in a general guide to neuropsychological assessment [[5](#_ENREF_5)] and treated as based on focused attention by others (eg, [[6](#_ENREF_6)]).
- Test such as trail making test B were treated as executive function markers, rather than attention/PS. Trail making test A was not used as a control by the formation of difference or ratio scores and was treated as a measure of attention/processing speed. Similar allocation of Trail making tests A and B to executive and processing speed domains has been endorsed by others (eg, [[7](#_ENREF_7)]).
- Letter (also known as phonemic) fluency was categorised as a measure of executive function and semantic (also known as category) fluency measures were categorized in the language domain. This is consistent with other studies (eg, [[8](#_ENREF_8)]), and with the psychometrically-based allocation of phonemic fluency to an executive function scale [[4](#_ENREF_4)].

*Calculation of domain scores: general approach*

In general, domain scores were formed as the sum (or average) of component test scores, adjusted for age, sex and education, and all interactions between these variables. The composite scores were then transformed to Z-scores, so that all domains had means of 0 and standard deviations (SDs) of 1 within each study.

However, when within a composite, there are one or more clusters of closely related tests, a standardised composite of adjusted scores for these tests are first formed, and then this combined with the adjusted scores of other tests (or composites, if any) in the domain (for example, the strongly inter-correlated multiple measures derived from the RAVLT were first combined, before combining this composite with the other tests to form a memory domain). This was done so that very similar multiple tests within a domain do not result in an over-representation of that construct in the final domain score. In some cases when the scales of very similar multiple tests are the same, in line with common practice, a composite of these tests is formed without first adjusting each test. (For example scores the separate fluency tests F, A and S would be simply added to form a total fluency score, FAS).

In this first set of analyses, in order to maximise the number of studies included, and the number of domains formed, no restriction was placed on the number of tests within each domain that were used to form the domain scores. The number of tests within each domain could vary between studies for a given domain, and within studies between different domains. Mean scores were calculated on the basis of all tests present in the domain. A domain score would be missing for a particular case if either there were no tests allocated to that domain in that study, or else all tests allocated to that domain were missing for that case.

Also, a policy was adopted of having a preference to maintaining the original distribution of scores, without transforming to more closely approximate the normal distribution. Extreme values were Winsorised only if more than 4 SDs from means, and distributions transformed to reduce skewness only if the original skewness was greater than about 3.

Steps in more detail:

1. Using OLS regression analysis, save standardised residuals for each test (controlling for age, sex and education, and all interactions).
2. Examine distribution of each adjusted variable and Winsorised to 4 SDs, or transform using appropriate function if skewness greater than about 3.
3. Reverse the scores (multiply by −1) if necessary so that more positive scores represent better performances.
4. If there are any groups of similar tests within each domain, form sum(s) of scores for each of these and transform to Z-scores.
5. Sum the above scores from individual tests, or derived from either groups of tests, to form domain composites, and transform these composites to Z-scores.

*Caveats for determining no or minimal functional impairment for cases with missing data*

Participants were classified as not functionally impaired if they had data for 4 or more items and were independent on all or all but 1 of these, or if they had data for 3 items and were independent on all of these. Other participants with data for only 3 items, and those with data for less than 3 items and not dependent for at least 2 were considered to have insufficient data and were not classified.

## *Calculation and comparison of standardized prevalence estimates*

The standardized prevalence estimate, Ps, for each study was calculated using the formula:

Ps = Σ Q_i_ P_i_ ; i = 1 to k;

Where:

k is the number of subsamples of the particular study (e.g. based on sex and age range groupings).

Q_i_ is the proportion of cases in i’th subsample in the reference distribution.

P_i_ is the proportion of cases with MCI in the i’th subsample in the study.

N_i_ is the number of cases in the i’th subsample of the study.

In order to test the null hypothesis of equality of standardized prevalence estimates across a number (m) of studies, the method described by Armitage et al. [[9](#_ENREF_9)] was used.

First, the standard error of measurement squared for the standardized prevalence estimate, Var(Ps), for each study was calculated:

Var(Ps) = Σ Q_i_^2^ P_i_(1 – P_i_)/N_i_ ; i = 1 to k.

Next a “weighted mean”, M, of the Ps’s across the m chosen studies was obtained:

M = (Σ w_i_ Ps_i_)/ (Σ w_i_); i = 1 to m.

Where:

m is the number of studies for which equality of Ps’s is being tested.

w_i_ = 1/Var(Ps) for study i, i.e. is equal to the reciprocal of the variance of i’th study, as obtained above.

A test statistic, G, is then calculated, that has a Chi-squared distribution, with degrees of freedom,

df = m - 1:

G = Σ w_i_ (Ps_i_ - M)^2^

##

## *Procedure for multiple imputation of missing age group data*

Imputation was done separately for each of the five types of MCI classifications. For each of the three age groups, values were generated for both the number of participants classified as having MCI and the total number of participants in the group. Sex was a covariate in the analyses. We used the predictive mean matching method of multiple imputation, in which expected values are first obtained conditional on the observed covariates (regression). Where values are initially missing, the method next identifies one or more neighbor observations which have similar estimated values. The observed value of the nearest neighbor is used to replace the missing value. This imputation method was implemented in the R-package “mice”, as outlined by van Buuren and Groothuis-Oudshoorn [[10](#_ENREF_10)].

**References**

1. Lezak MD, Howieson DB, Loring DW. Neuropsychological Assessment. 4 ed. New York, NY: Oxford University Press; 2004.

2. Strauss E, Sherman EMS, Spreen O. A Compendium of Neuropsychological Tests: Administration, Norms, and Commentary. 3rd ed. New York, NY: Oxford University Press; 2006.

3. Weintraub S, Salmon D, Mercaldo N, Ferris S, Graff-Radford NR, Chui H, et al. The Alzheimer's Disease Centers' Uniform Data Set (UDS): the neuropsychologic test battery. Alzheimer Dis Assoc Disord 2009;23:91-101.

4. Mungas D, Reed BR, Kramer JH. Psychometrically matched measures of global cognition, memory, and executive function for assessment of cognitive decline in older persons. Neuropsychology 2003;17:380-92.

5. Hebben N, Milberg W. Essentials of Neuropsychological Asessment. 2nd ed. Hoboken, NJ: John Wiley & Sons; 2009.

6. Babiloni C, Cassetta E, Binetti G, Tombini M, Del Percio C, Ferreri F, et al. Resting EEG sources correlate with attentional span in mild cognitive impairment and Alzheimer's disease. Eur J Neurosci 2007;25:3742-57.

7. Lim J, Oh IK, Han C, Huh YJ, Jung IK, Patkar AA, et al. Sensitivity of cognitive tests in four cognitive domains in discriminating MDD patients from healthy controls: a meta-analysis. Int Psychogeriatr 2013;25:1543-57.

8. Ganguli M, Snitz BE, Lee CW, Vanderbilt J, Saxton JA, Chang CC. Age and education effects and norms on a cognitive test battery from a population-based cohort: the Monongahela-Youghiogheny Healthy Aging Team. Aging Ment Health 2010;14:100-7.

9. Armitage P, Berry G, Matthews JNS. Statistical Methods in Medical Research. 4th ed. Massachusetts: Blackwell; 2002:215-6.

10. van Buuren S, Groothuis-Oudshoorn K. mice: Multivariate Imputation by Chained Equations in R. J Stat Softw 2011;45:1-67.
