# Supplementary material for: The Prevalence of Mild Cognitive Impairment in Diverse Geographical and Ethnocultural Regions: The COSMIC Collaboration
Source: PLoS One. 2015 Nov 5;10(11):e0142388. doi: 10.1371/journal.pone.0142388 (PMC4634954; doi:10.1371/journal.pone.0142388)
Supplement: S2 Text — (DOCX) [file pone.0142388.s021.docx]

**S2 Text. Supplementary Results**

*1. Effects of controlling for education on differences in MCI prevalence estimates among studies*

In regression models controlling for age and sex, the Wald statistic for a categorical study variable decreased from 65.868 (*P* < .001) to 60.877 (*P* < .001) when education was included. Further regressions for all possible study pairs that either included or did not include education produced results reasonably consistent with this. For study pairs where there was a significant difference in MCI prevalence estimates, in most cases (80%) controlling for education negated the difference or reduced its magnitude. However, there were a small number of cases where controlling for education had the opposite effect.
